# Acute HIV Infection and ART Response: Insights into T Cell Subsets, Activation, Exhaustion, and Blood and GALT HIV Reservoir

**DOI:** 10.3390/v17101381

**Published:** 2025-10-16

**Authors:** Soraia Santana de Moura, Diogo Gama Caetano, Monick Lindenmeyer Guimarães, Rayana Katylin Mendes da Silva, Natasha Cabral, Simone da Costa Cruz Silva, Marcelo Ribeiro-Alves, Sylvia L. M. Teixeira, Ingebourg Georg, Desirée Vieira Gomes dos Santos, Sandro Nazer, Rafael Teixeira Fraga, Brenda Hoagland, Larissa Villela, Beatriz Gilda Jegerhorn Grinsztejn, Valdiléa Gonçalves Veloso, Fernanda Heloise Côrtes, Sandra W. Cardoso

**Affiliations:** 1 Laboratório de Pesquisa Clínica em IST e AIDS, Instituto Nacional de Infectologia Evandro Chagas, Fundação Oswaldo Cruz, Rio de Janeiro 21040-900, Brazil; soraia.moura@ini.fiocruz.br (S.S.d.M.); marcelo.ribeiro@ini.fiocruz.br (M.R.-A.); desiree.santos@ini.fiocruz.br (D.V.G.d.S.); sandro.nazer@ini.fiocruz.br (S.N.); brenda.hoagland@ini.fiocruz.br (B.H.); laramvillela@gmail.com (L.V.); gbeatriz@ini.fiocruz.br (B.G.J.G.); valdilea.veloso@ini.fiocruz.br (V.G.V.); 2 Laboratório de Aids e Imunologia Molecular, Instituto Oswaldo Cruz, Fundação Oswaldo Cruz, Rio de Janeiro 21040-900, Brazil; diogocaetano@aluno.fiocruz.br (D.G.C.); monicklg@ioc.fiocruz.br (M.L.G.); sylvia@ioc.fiocruz.br (S.L.M.T.); 3 Laboratório de Hanseníase, Instituto Oswaldo Cruz, Fundação Oswaldo Cruz, Rio de Janeiro 21040-900, Brazil; natasha.cabral@ioc.fiocruz.br; 4 Plataforma de Laboratório Multiusuário, Instituto Nacional de Infectologia Evandro Chagas, Fundação Oswaldo Cruz, Rio de Janeiro 21040-900, Brazil; simone.silva@ini.fiocruz.br; 5 Laboratório de Imunologia, Instituto Nacional de Infectologia Evandro Chagas, Fundação Oswaldo Cruz, Rio de Janeiro 21040-900, Brazil; ingebourg.georg@ini.fiocruz.br

**Keywords:** acute infection, early treatment, activation, exhaustion, Fiebig, CD4/CD8 ratio, GALT, HIV, cure

## Abstract

Investigating immunological and viral reservoir dynamics in blood and GALT during acute HIV phase advances understanding of HIV persistence. Dynamics of T cells and HIV reservoirs immediately after early ART require further investigation. We evaluated the ART impact at 12 (M12) and 24 months (M24) on immunological, virological and reservoir markers of 24 participants starting ART at Fiebig ≤ V (Baseline = D0) in a Brazilian cohort. We measured the frequency of T cell activation, exhaustion, memory subsets, Th17 and pTfh cells by flow cytometry and quantified total HIV DNA by qPCR in PBMC and GALT. Most participants were cisgender MSM (95.9%), with a median age of 27 years (IQR 25–36). At enrollment (D0), four participants used triple ART as PEP, and two were under oral PrEP and they exhibited higher CD4/CD8 ratios. Higher CD4/CD8 ratios were also observed in participants classified as Fiebig I to III. A total of 92% achieved viral suppression at M12 and 96% at M24. CD4 counts rose from 646 to 861 cells/mm^3^, and the CD4/CD8 ratio improved from 0.76 to 1.24 (*p* < 0.01). HIV DNA in PBMCs decreased 4-fold by M12 and 61-fold by M24, with 50% of participants reaching undetectable levels by M24 (*p* < 0.01). In GALT, undetectable HIV DNA increased from 27% at D0 to 75% at M12. HIV DNA in PBMCs and GALT correlated with plasma VL, while the CD4/CD8 ratio was inversely linked to PBMC reservoirs (rho = −0.66; *p* < 0.05). Early ART reduced activated CD8^+^ T cells (*p* < 0.05) but had minimal effects on CD4^+^ T cells or exhaustion markers. By M24, CD8^+^ TCM increased, and CD8^+^ TEF decreased (*p* < 0.01), while Th17 and pTfh levels remained stable. Early ART led to viral suppression and immune restoration, and influenced reservoir dynamics. The CD4/CD8 ratio was shown to be a key marker of early treatment success. Since a quarter of the participants were identified while initiating PrEP/PEP, it is important to consider the acute phase window according to vulnerability. Recent PEP/PrEP use often excludes participants from clinical trials on bNAbs and therapeutic vaccines targeting viral reservoirs during the acute phase of HIV. Since these are the populations that may benefit from these strategies, larger studies including those people are needed.

## 1. Introduction

Today, a single once-daily antiretroviral therapy (ART) tablet containing three drugs results in viral control in nearly all people living with HIV (PLHIV) who can access and adhere to daily regimens [1]. Despite its effectiveness, ART is not curative and requires lifelong administration. Treatment success hinges on sustained viral suppression, highlighting the importance of strict adherence. However, even the most effective therapeutic regimens do not eradicate the virus. Once ART is interrupted, rapid viral rebound within days to weeks is observed [2,3,4], and the virus remains indefinitely in latent reservoirs, which represents the greatest challenge to HIV eradication [5,6,7].

Acute or early HIV infection (AEHI) manifests with viral and immunological characteristics that determine the course of the disease, with damage to the immunological components and establishment of viral reservoirs already observed in this phase [8,9,10,11]. The mechanisms driving viral reservoir formation and heightened immune activation during HIV-1 infection are not completely understood. Most infections occur through sexual contact, involving genital and/or rectal mucosa exposure [8,9]. Once the virus crosses the epithelial barrier, the gastrointestinal mucosa is commonly the primary site for HIV-1 replication [10].

In recent years, research focusing on anatomical reservoir sites like gut-associated lymphoid tissue (GALT) has been valuable. GALT harbors many activated CD4^+^ T cells, which make this site crucial to disease pathogenesis [11,12,13,14]. However, there is a paucity of studies evaluating GALT samples in HIV acute infection. Early ART in AEHI may preserve CD4^+^ cells in GALT, including Th17 cells, which contribute to GALT homeostasis and protection [15,16]. Th17 cells are identified mainly by expression of IL-17, but other markers such as CCR6 and CD161 are also useful. They produce IL-17, IL-21, and IL-22, crucial for tissue regeneration, pathogen defense, and intestinal barrier maintenance [17,18,19,20,21]. Persistent CD4^+^ T cell activation may drive activated CD4^+^ T cells toward the Th17 phenotype, heightening disease progression risk despite virological control [22].

In chronic HIV infection, there is a notable decline of Th17 cells in the mucosa, leading to microbial translocation and sustained immune activation despite antiretroviral therapy [22,23]. While Th17 cells are preserved during Fiebig (F) stages I/II, their numbers and function decrease during stage F III, correlating with markers of local and systemic immune activation. Early ART initiation during stages F I/II prevents the loss of mucosal Th17 cells and their function, whereas initiation during stage FIII restores cell numbers but not their full functionality. Additionally, starting ART early during stages F I/II completely reverses initial mucosal and systemic immune activation. These findings underscore the early loss of Th17 cells in acute HIV infection and advocate for further research into early ART initiation during acute HIV to better understand how it preserves mucosal Th17 cells function [24].

During chronic HIV infection, immune activation is marked by increased T and B lymphocytes with an activated phenotype and higher levels of pro-inflammatory cytokines and chemokines. CD38 and/or HLA-DR expression on T cells is a significant prognostic factor for HIV-1 infection [25] and is linked to disease development, severity, and mortality, likely affecting the viral reservoir [26]. Moreover, delayed treatment is related to sustained CD8^+^ T cell activation [24,27].

Persistent viral replication leads to T cell exhaustion, which impairs immune function and correlates positively with disease progression [26]. Exhausted HIV-1-specific T cells show functional and proliferative loss, contributing to viral persistence. In chronic HIV infection, cellular exhaustion not only affects HIV-specific CD4^+^ and CD8^+^ T cells, but also disrupts overall T cell dynamics [28]. The expression of key markers of exhaustion, including PD-1 and Tim-3, is elevated in HIV infection and positively correlated to disease progression [26,29].

However, significant advances in the understanding of immune responses in HIV have emerged from studies of Post-treatment Controllers (PTCs), who are rare individuals able to control viremia after interrupting ART, offering critical insights into potential HIV cure strategies. The VISCONTI cohort focuses on people living with HIV (PLHIV) who initiated early ART and sustained viral control after stopping treatment, underscoring the importance of early intervention in achieving long-term infection control [30]. Recent findings from the CHAMP study (Control of HIV After Antiretroviral Medication Pause) indicate that PTCs have a unique set of immunological profiles compared to non-controllers, highlighting their ability to maintain lower inflammation and T cell activation, especially in CD4^+^ T cells [4,31]. This capability may be related to memory CD8^+^ T cells after treatment interruption, which have a stronger antiviral potential if treatment is initiated within four weeks of infection (as was the case for most participants in the VISCONTI study), suggesting a window of opportunity to achieve HIV infection remission [32]. A subset of peripheral Tfh (pTfh) cells was shown to be preserved in HIV controllers [33], which may also have an important role in the context of early treatment in acute infection.

Investigating immunological components and viral reservoir dynamics in both blood and GALT in AEHI patients treated early can significantly advance the understanding of HIV persistence. This study describes virological and immunological markers from a Brazilian acute HIV infection cohort, contributing to ongoing and future HIV research on prevention, treatment, and cure strategies.

## 2. Materials and Methods

### 2.1. Study Design

This is a prospective ongoing cohort study at Instituto Nacional de Infectologia Evandro Chagas–INI/Fiocruz, aiming to evaluate clinical, virological, and immunological parameters of participants initiating ART during acute/early HIV infection. INI is a major HIV referral-testing center and the largest pre-exposure prophylaxis (PrEP), and ART provider in Rio de Janeiro, providing services for sexual and gender minorities. HIV post-exposure prophylaxis (PEP) is provided 24/7. This cohort has been enrolling participants seeking INI services since August 2013. Immediate ART initiation for any individual diagnosed with HIV is offered according to the Brazilian ART Guidelines [34].

Acute HIV infection was defined as a non-reactive third-generation HIV rapid test followed by a reactive HIV antigen/antibody combination assay, or a detectable HIV RNA pooled testing, subsequently confirmed with an individual HIV RNA test. Early HIV infection was defined as a reactive HIV serology and a documented HIV negative serology within the prior six months or a reactive Western blot lacking p31 (pol) reactivity. All enrolled participants were classified according to the Fiebig stage [35] at ART initiation.

HIV RNA testing was performed for all negative HIV rapid test results in the following situations: report of condomless anal sex (with or without suspicious signs and symptoms of acute viral infection) >72 h and ≤30 days before PEP initiation (day-1 PEP) and one week later (day-7 PEP), before PrEP start or re-start and in the presence of an STI (syphilis, hepatitis B, hepatitis C, and CTNG).

From December 2013 to August 2024, 111 participants presenting acute or early HIV infection (AEHI) characteristics were enrolled in the cohort. Of these, 50 participants who were at the Fiebig stage ≤V at ART initiation between December 2013 and December 2022 were selected for this study.

**Figure 1:**
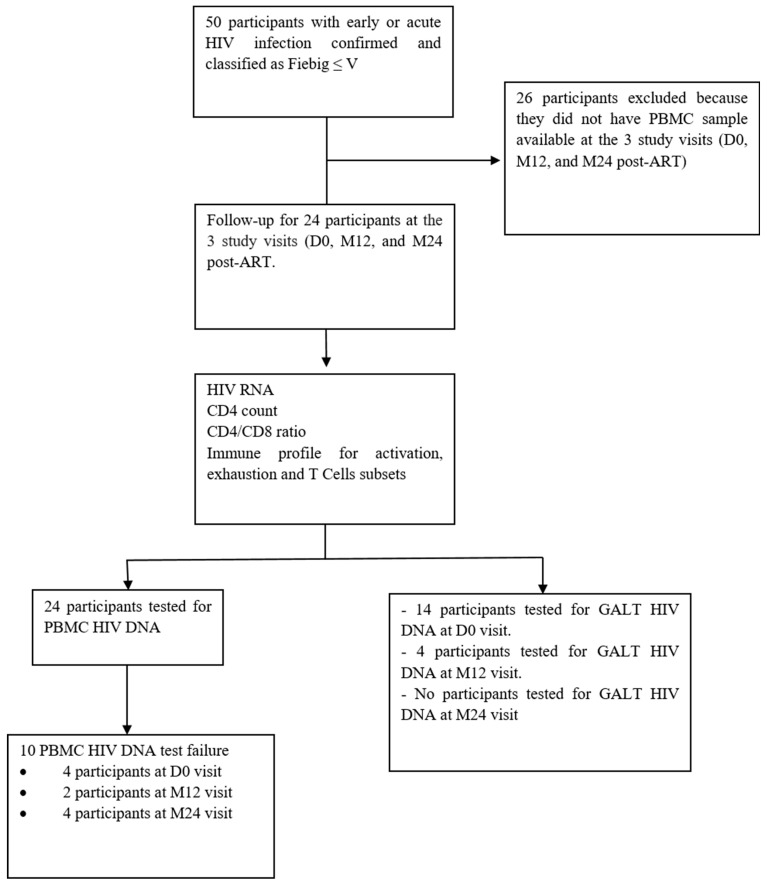
Flowchart of the study. D0: Day 0 or pre-ART visit; M12: month 12; M24: month 24; GALT: gut-associated lymphoid tissue; PBMC: peripheral blood mononuclear cells.

Due to the inherent difficulty in obtaining tissue samples, only 14 of the 24 selected participants had rectal tissue acquired by biopsy in at least 1 visit (Figure 1).

All participants provided written informed consent, and the study was approved by the ethics committee of INI-Fiocruz (CAAE 36859614.8.0000.5262).

### 2.2. HIV Viral Load CD4 and CD8 T Cell Count

The HIV viral load was measured in plasma using the Abbott Real Time HIV-1 kit (Abbott Molecular, Des Plaines, IL, USA), according to the manufacturer’s recommendations. The lower detection limit was 40 copies/mL and the highest limit was 10 million copies/mL.

Absolute CD4^+^ and CD8^+^ T cell counts were determined in whole blood through flow cytometry using the Multitest TruCount Kit and the Multiset software (Version 2.2) of the BD FACSCalibur or FACSVia flow cytometer (Version 2.0) according to the manufacturer’s recommendations.

### 2.3. Blood Collection and PBMC Isolation

PBMCs were isolated from whole blood using Histopaque-1077 (Sigma-Aldrich, St. Louis, MO, USA) by density gradient centrifugation, cryopreserved in fetal bovine serum (FBS) with 10% DMSO at a concentration of 5–15 × 10^6^ cells/cryovial, and stored in liquid nitrogen until use.

### 2.4. T Cell Activation, Exhaustion, Memory Subsets, and pTfh Cells

Cryopreserved PBMCs were thawed and rested overnight in RPMI 1640 (Gibco, Waltham, MA, USA) supplemented with 10% of FBS (Gibco, Waltham, MA, USA) at 37 °C, with 5% of CO_2_ and under controlled humidity.

After resting, cells were counted and stained with LIVE/DEAD (Thermo Fisher Scientific, Waltham, MA, USA) to exclude dead cells. PBMC were subsequently stained with monoclonal antibodies anti-CD3 BV510, anti-CD8 APC, anti-CD4 PECF594, and anti-HLA-DR APC eFluor780, and anti-CD38 FITC for the evaluation of activation, anti-PD-1 BV785 for exhaustion, anti-CCR7 PE and anti-CD45RA eFluor450 T cells subsets, Naïve T cells (TN: CD45RA^+^ CCR7^+^), Central Memory T cells (TCM: CD45RA^−^ CCR7^+^), Effector Memory T cells (TEM: CD45RA^−^ CCR7^−^), and Effector T cells (TEF: CD45RA^+^ CCR^−^ and anti-CXCR5 BB700 for pTfh subset. After staining, the samples were fixed using PBS-PFA 1% solution and acquired using a BD FACSymphony A5 flow cytometer (BD Biosciences, Franklin Lakes, NJ, USA). Analyses were performed with FlowJo v.10.8.1 (TreeStar, Ashland, OR, USA). The gate strategy is described in Supplementary Figure S1.

### 2.5. Th17 Cells and IL-22^+^ Cells

To access the frequency of Th17 cells, thawed PBMCs were suspended in RPMI 1640 medium (Gibco, Grand Island, NY, USA) with 10% FBS (Gibco, Grand Island, NY, USA). One million cells were stimulated with 5 ng/mL phorbol myristate acetate (PMA) plus 1 μg/mL ionomycin (Sigma-Aldrich, St. Louis, MO, USA) for 6 h at 37 °C with 5% CO_2_. After 30 min of stimulus, brefeldin A (5 μg/mL; Sigma-Aldrich, St. Louis, MO, USA) and GolgiStop (5 μL/mL; BD Biosciences, Franklin Lakes, NJ, USA) were added. At the end of stimulation, cells were washed with PBS and stained with LIVE/DEAD (Thermo Fisher Scientific, Waltham, MA, USA) to exclude dead cells. Cells were then stained with anti-CCR6 BV650, anti-CD161 PERCPCY-5, anti-CD4 BV785, anti-CD8 APC-H7, and anti-CD3-BV510, fixed with 4% PFA, permeabilized using PERM WASH solution (BD Biosciences, Franklin Lakes, NJ, USA) and labeled with anti-IL-17 PECY-7 and anti-IL-22 APC. Samples were acquired on a BD FACSymphony A5 flow cytometer (BD Biosciences, Franklin Lakes, NJ, USA) and subsequently analyzed using FlowJo v.10.8.1 (TreeStar, Ashland, OR, USA). The gate strategy is described in Supplementary Figure S1.

### 2.6. DNA Extraction and HIV DNA Quantitation

For isolation of PBMC DNA, cryopreserved PBMCs at concentrations between 10 and 15 million cells/mL were thawed and treated with Proteinase K (100 μg/mL, Invitrogen, Carlsbad, CA, USA) at 55 °C under overnight agitation, followed by inactivation of the enzyme by heating at 95 °C for 5 min, and stored at −20 °C until use.

For isolation of tissue DNA, the rectal biopsies obtained by colonoscopy or flexible rectosigmoidoscopy and stored in RNA later (−80 °C freezer) were thawed, crushed with a scalpel, and placed in TRIzol Reagent (Invitrogen, Carlsbad, CA, USA) for cell lysis. Chloroform was added to the sample, which was incubated for 3 min and centrifuged at 12,000× *g* at 4 °C for 15 min. The aqueous phase was then discarded, and ethanol 100% (Sigma-Aldrich, St. Louis, MO, USA) was added and centrifuged at 2000× *g* at 4 °C, and the pellet was resuspended in sodium citrate (0.1 M–10% ethanol (Sigma-Aldrich, St. Louis, MO, USA) and then incubated for 30 min. The tube was then centrifuged, and the pellet was resuspended in 75% ethanol and incubated for 10 to 20 min. The tube was centrifuged, and the pellet was left to dry for 5 to 10 min and resuspended in NaOH 8 mM (Sigma-Aldrich, St. Louis, MO, USA) until complete dissolution. Then, the sample was centrifuged again and 1 mM EDTA was added and stored at −20 °C until use.

For Total HIV DNA quantitation, 15 µL of DNA suspension were used to pre-PCR as previously described by Chomont et al. [36]. Simultaneously, a standard curve varying from 3 to 300 thousand cells, obtained from the serial dilution of DNA from cells of the ACH-2 lineage was amplified under the same conditions. The pre-amplified DNA from the samples and the curve were then diluted in the proportion 1:2 and used to carry out two qPCR reactions using TaqMan Universal Master Mix II (Thermo Fisher Scientific, Waltham, MA, USA), one containing CD3 and another containing the viral DNA. The reactions were performed using the QuantStudio™ 5 Real-Time PCR System (Thermo Fisher Scientific, Waltham, MA, USA), and the results obtained for HIV were normalized based on CD3 quantification and expressed as the number of HIV copies per million cells.

### 2.7. Statistics

For descriptive analysis, data were presented as medians with interquartile ranges (IQRs), representing the spread between the 75th and 25th percentiles, or as frequencies (percentages) for numerical-continuous and categorical data, respectively, and comparisons among visits (i.e., D0, M12, and M24) were tested by either Kruskal–Wallis (numerical-continuous variables) or chi-squared (discrete-nominal variables) tests. Skewed numerical-continuous variables underwent log transformation. Linear multiple mixed-effect models were used to account for time dependencies within patients (i.e., patient identities were included as a random effect). The Tukey Honest Significant Difference (HSD) method was used to correct *p*-values by the number of pairwise comparisons. Correlation analyses were conducted using Pearson’s coefficients. *p* ≤ 0.05 was considered statistically significant. R software version 4.1.2, packages ‘lme4’, ‘emmeans’, and their dependencies were used to perform the statistical analyses.

## 3. Results

### 3.1. Cohort Characteristics

Among the 24 participants, 23 (95.9%) were cisgender men who have sex with men (MSM) and one (4.1%) was a transgender woman. The median age was 27 years (25th-75th = 25–36) (Table 1). At visit D0, four participants (16.6%) were under a triple ART regimen prescribed as PEP, two participants (8.3%) were under TDF/FTC PrEP, and 18 (75%) were not taking antiretroviral medications (Supplementary Table S1). Among the 24 participants, four were identified AEHI cases while seeking PEP within 72 h of sexual exposure.

### 3.2. HIV Viral Load CD4 and CD8 T Cell Count

**Figure 2:**
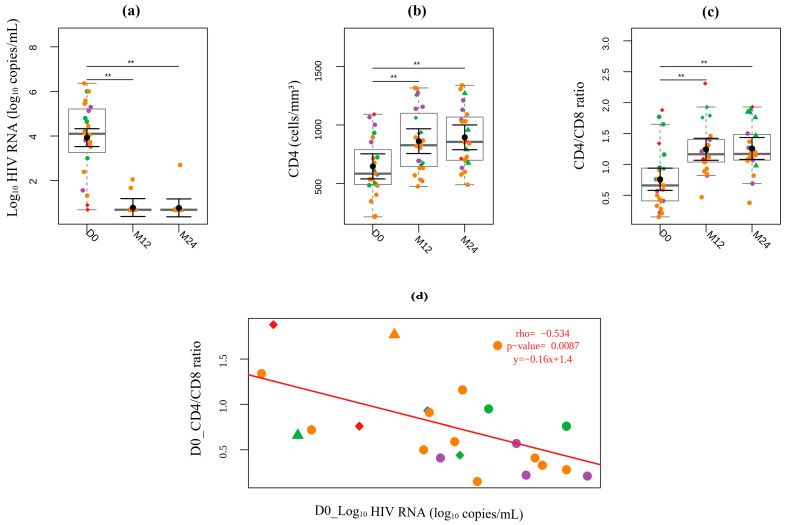
Longitudinal analysis of viral load and T cell count. (**a**) HIV viral load, (**b**) CD4 T Cell Count, and (**c**) CD4/CD8 Ratio at D0, M12, and M24. Fiebig stages are identified by colors as follows: red (I), blue (II), green (III), purple (IV), and orange (V). Participants who began ART prescribed as post-exposure prophylaxis (PEP) before the diagnosis are represented by the diamond symbol, while those identified while seeking pre-exposure prophylaxis (PrEP) at the time of diagnosis are represented by triangle symbols. For black error bars, central dots represent mean marginal estimates and parallel lines indicate the upper and lower 95% confidence interval limits. Gray boxplots represent the boxplot of the sampled distribution. (**d**) Correlation analysis of CD4/CD8 ratio and HIV RNA at D0. *p*-values were generated from mixed linear regression analysis and correlations were analyzed using Pearson’s correlation test. ** indicates *p* < 0.01.

The mean viral load at D0 was 3.9 log_10_ copies per mL (3.5–4.3) (Table 1), and 22 participants (92%) achieved undetectable viral load at M12 visit (Figure 2a). One participant presented 460 copies/mL at M12 and undetectable VL at M24 visit, while another presented virological failure at M12 and M24 visits (HIV RNA > 1000 copies/mL) related to poor adherence, despite achieving suppression at three months after TARV initiation.

The mean CD4 count at the D0 visit was 646 cells/mm^3^ (540–754) (Table 1), with seven participants (29%) presenting T CD4 counts below 500 cells/mm^3^. At 12 months post-treatment, the mean CD4 count increased significantly to 861 cells/mm^3^ (756–967; *p* < 0.001) and remained elevated at 24 months [895 cells/mm^3^ (789–1000); *p* < 0.001]. At both follow-up visits, nearly all participants (96%; n = 23) had at least 500 CD4^+^ T cells/mm^3^ (Figure 2b).

At D0, the mean CD4/CD8 ratio was 0.76, with 18 (75%) participants having a CD4/CD8 ratio < 1.0 (Figure 2c). Five participants showed a CD4/CD8 ratio greater than 1 at the D0 visit, two of whom were Fiebig I and three Fiebig III. Among participants who presented a CD4/CD8 ratio >1, four (80%) were earlier exposed to ART (PEP = 3, PrEP = 1) (Supplementary Table S1). The use of ART consistently improved the CD4/CD8 ratio over time (Figure 2c); the mean of CD4/CD8 ratio at M12 was 1.24 (1.06–1.42) and 1.26 (1.08–1.44) at M24. Additionally, the CD4/CD8 ratio negatively correlated with viral load levels at D0 (r = −0.54; *p* = 0.009) (Figure 2d).

### 3.3. Quantification of Total HIV DNA

To evaluate the impact of early treatment on the viral reservoir, we measured total HIV DNA in PBMC and GALT. Twenty participants had PBMC samples available for viral reservoir analysis at D0. Of these, eight participants (40%) had undetectable levels of HIV DNA at D0: four were on ART before confirmation of AHI (triple therapy prescribed as PEP); two had prior exposure to PrEP before initiating triple combination ART; one collected blood samples at the visit three days after initiating ART; and one had no prior antiretroviral use.

**Figure 3:**
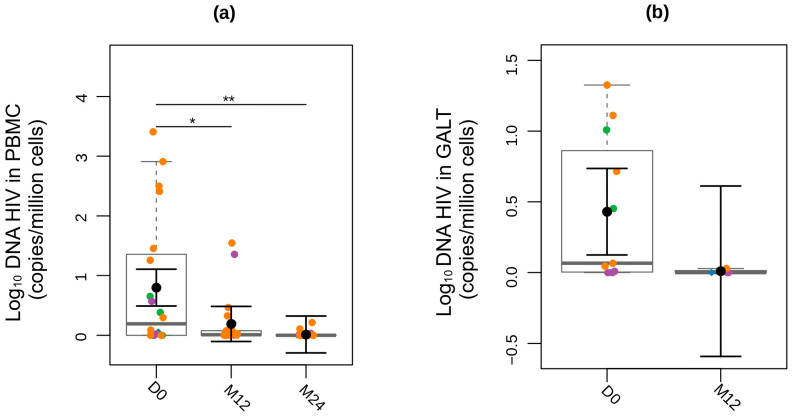
Longitudinal analysis (**a**) Peripheral Blood Mononuclear Cells (PBMCs) and (**b**) Gut-Associated Lymphoid Tissue (GALT) harboring total HIV DNA at D0, M12, and M24 Fiebig stages are identified by colors as follows: red (I), blue (II), green (III), purple (IV), and orange (V). Participants who began ART prescribed as post-exposure prophylaxis (PEP) before the diagnosis are represented by the diamond symbol, while those identified while seeking pre-exposure prophylaxis (PrEP) at the time of diagnosis are represented by triangle symbols. For black error bars, central dots represent mean marginal estimates and parallel lines indicate the upper and lower 95% confidence interval limits. Gray boxplots represent the boxplot of the sampled distribution. The *p*-values were generated from mixed linear regression analysis. * indicates *p* < 0.05; ** indicates *p* < 0.01.

There was a reduction in total HIV DNA in PBMCs after treatment at both M12 (*p* = 0.012) and M24 (*p* = 0.001) visits. The mean viral reservoir level at D0 was 0.80 Log_10_ copies per million cells (0.49–1.1 log_10_ copies/million cells), compared with 0.19 log_10_ copies per million cells at M12 (−0.1–0.48 log_10_ copies/million cells) and 0.01 Log_10_ copies per million cells at M24 (−0.29–0.32 log_10_ copies/million cells). Ten participants (50%) achieved undetectable levels of HIV DNA in PBMCs at the M24 visit (Figure 3a).

Eleven participants had GALT samples available for viral reservoir analysis at D0 (Figure 3b). Three of them had undetectable levels of HIV DNA, and the mean viral reservoir at D0 was 0,43 copies/million cells (0.12–0.74 log_10_ copies/million cells). Only four participants had GALT samples available for analysis at the M12 visit, and three of them had undetectable levels of HIV DNA (Figure 3b). No samples were available at the M24 visit. We observed a trend toward a positive correlation between HIV DNA levels in GALT and PBMCs (Supplementary Figure S2a).

We identified a positive correlation between HIV DNA levels and plasma viral load, with PBMC quantification showing a moderate positive correlation (rho = 0.52, *p* < 0.02) and GALT showing a strong positive correlation (rho = 0.65, *p* = 0.03) (Supplementary Figure S2b,c). The CD4/CD8 ratio appears to be a significant marker in our study population, exhibiting a negative correlation with the viral reservoir in PBMC at all visits: D0 (rho = −0.63; *p* = 0.004), M12 (rho = −0.52; *p* = 0.018), and M24 (rho = −0.54; *p* = 0.014) (Supplementary Figure S2d–f). This correlation was not observed for GALT. However, HIV DNA in GALT showed a strong negative correlation with CD4 count at D0 (r = −0.72; *p* = 0.012), a pattern not seen in PBMC (Supplementary Figure S2g).

### 3.4. T Cell Activation

**Figure 4:**
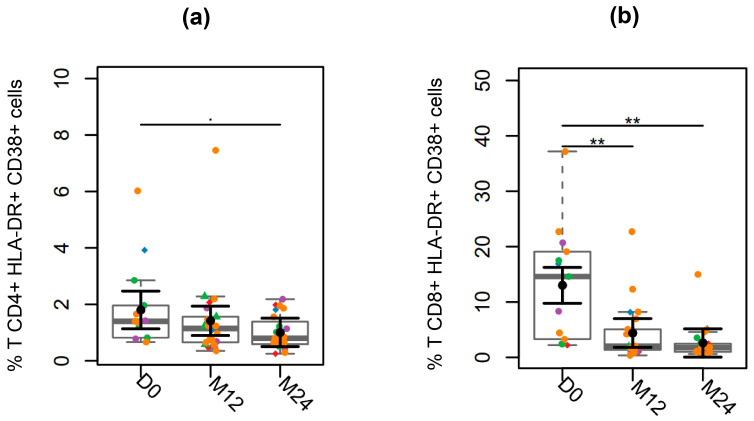
Longitudinal analysis T cell Activation. (**a**) CD4^+^ HLA-DR^+^ CD38^+^ and (**b**) CD8^+^ HLA-DR^+^ CD38^+^ T cells. Fiebig stages are identified by colors as follows: red (I), blue (II), green (III), purple (IV), and orange (V). Participants who began ART prescribed as post-exposure prophylaxis (PEP) before the diagnosis are represented by the diamond symbol, while those identified while seeking pre-exposure prophylaxis (PrEP) at the time of diagnosis are represented by triangle symbol. For black error bars, central dots represent mean marginal estimates and parallel lines indicate the upper and lower 95% confidence interval limits. Gray boxplots represent the boxplot of the sampled distribution. *p*-values were generated from mixed linear regression analysis and correlations were analyzed using Pearson’s correlation test. ** indicates *p* < 0.01.

To investigate the impact of ART on T cell activation, we analyzed the frequency of cells expressing HLA-DR and CD38. After initiating ART, we observed a trend toward a decrease in the frequency of activated CD4^+^ T cells (Figure 4a). The mean of activated cells was 27% lower at M12 and 55% lower at M24 compared to D0. Notably, two participants—one who was exposed to oral PrEP and another who started ART as PEP—had HLA-DR^+^CD38^+^ cell frequencies at D0 that were nearly 50% lower than the group mean (0.82% and 0.74%, respectively, compared to the group mean of 1.8%).

The reduction in the frequency of the activated CD8^+^ T cell after initiating treatment was more pronounced, with significant decreases observed at M12 and M24 compared to D0 (*p* < 0.001 for both) (Figure 4b). This trend was also evident in the two participants mentioned earlier, whose activation profiles were substantially below the mean (2.4% and 2.2%, compared to the group mean of 13%). Additionally, we observed a strong positive correlation between the frequency of activated T cells at D0 and the level of the viral reservoir at M24 (CD4^+^: rho = 0.77; *p* = 0.006; CD8^+^: r = 0.68; *p* = 0.02). Furthermore, there was a significant positive correlation between the level of T cell activation at M12 and the viral reservoir at M12 (CD4^+^: rho = 0.73; *p* = 0.0003; CD8^+^: rho = 0.45; *p* = 0.04) and M24 (CD4^+^: rho = 0.80; *p* < 0.0001; CD8: r = 0.64; *p* = 0.004). These correlations were not observed in GALT (Supplementary Figure S3).

### 3.5. T Cell Exhaustion

**Figure 5:**
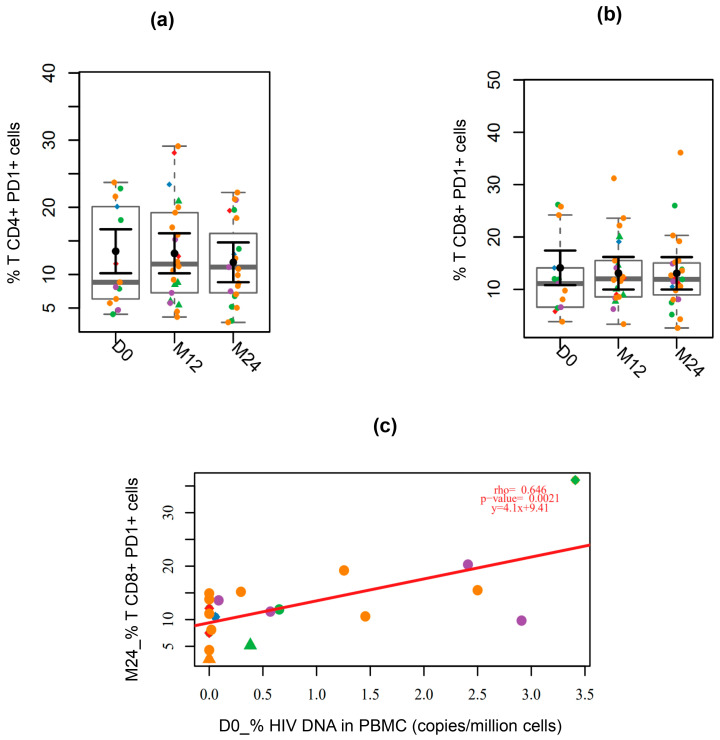
Longitudinal analysis of T cell exhaustion. (**a**) CD4^+^ PD-1^+^ T cells and (**b**) CD8^+^ PD-1^+^ T cells. (**c**) Correlation between expression of PD-1 at M24 and HIV reservoir in PBMC at baseline. Fiebig stages are identified by colors as follows: red (I), blue (II), green (III), purple (IV), and orange (V). Participants who began ART prescribed as post-exposure prophylaxis (PEP) before the diagnosis are represented by the diamond symbol, while those identified while seeking pre-exposure prophylaxis (PrEP) at the time of diagnosis are represented by triangle symbols. For black error bars, central dots represent mean marginal estimates and parallel lines indicate the upper and lower 95% confidence interval limits. Gray boxplots represent the boxplot of the sampled distribution. *p*-values were generated from mixed linear regression analysis and correlations were analyzed using Pearson’s correlation test.

The frequency of T cells expressing the exhaustion marker PD-1 was not significantly different between the analyzed visits in either CD4^+^ or CD8^+^ T cells (Figure 5a and Figure 5b, respectively). Notably, the participant with the highest level of PD-1 expression on CD4^+^ T cells at month 24 (M24) also exhibited the highest level of viral DNA in PBMCs at that visit and was among the five individuals with detectable viral DNA after 24 months of treatment (Supplementary Figure S3). Similarly, the individual with the highest level of CD8^+^ T cell exhaustion at baseline (D0) also had detectable viral DNA at M24. Furthermore, participants with higher levels of viral reservoir in PBMCs before treatment initiation showed increased PD-1 expression on CD8^+^ T cells at M24, with a significant positive correlation observed between baseline HIV DNA levels and the frequency of PD-1^+^ CD8^+^ T cells at month 24 (rho = 0.646; *p* = 0.005) (Figure 5c).

### 3.6. CD4 and CD8 T Cells Subsets

**Figure 6:**
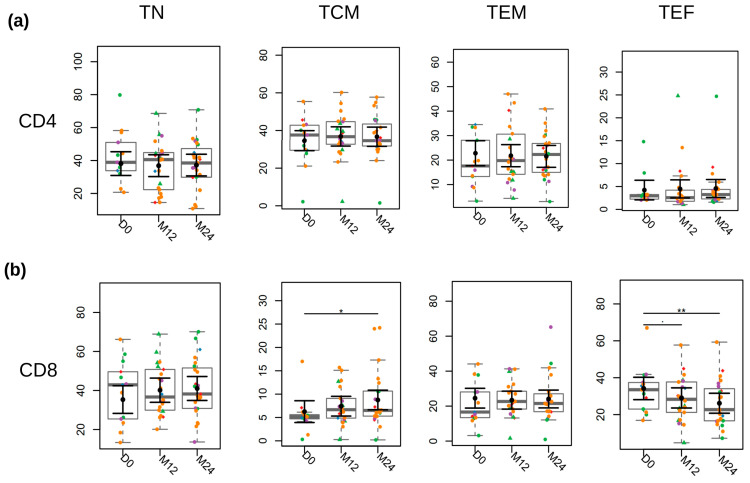
Longitudinal Analysis of the T cell substes. (**a**) CD4^+^ and (**b**) CD8^+^ T cell subsets. TN: Naive T cells, TCM: Central Memory T cells, TEM: Effector Memory T cells and TEF: Effector T cells. Fiebig stages are identified by colors as follows: red (I), blue (II), green (III), purple (IV), and orange (V). Participants who began ART prescribed as post-exposure prophylaxis (PEP) before the diagnosis are represented by the diamond symbol, while those identified while seeking pre-exposure prophylaxis (PrEP) at the time of diagnosis are represented by triangle symbols. For black error bars, central dots represent mean marginal estimates and parallel lines indicate the upper and lower 95% confidence interval limits. Gray boxplots represent the boxplot of the sampled distribution. *p*-values were derived from mixed linear regression analysis. * indicates *p* < 0.05; ** indicates *p* < 0.01.

We analyzed CD4^+^ and CD8^+^ T cell subsets to investigate the impact of early ART initiation on the frequency of naïve, memory, and effector cells. The frequency of the different CD4^+^ T cells subsets was similar in all visits (Figure 6a). After two years of treatment, we observed no differences in the frequency of CD8^+^ TN (naive) and TEM (effector memory) cells, but a significant increase in TCM (central memory) cells and a reduction in the frequency of TEF (effector) cells (Figure 6b).

We also assessed the frequency of pTfh cells (CD4^+^ CXCR5^+^) to understand their behavior in early treated AHI. We found no significant differences between study visits prior to ART use or Fiebig stages (Supplementary Figure S4). The frequency of Th17 cells were also evaluated, due to the limitation of the number of cells, this analysis was performed only at M12 and M24. We did not observe significant differences in Th17 cell frequencies between M12 and M24. However, there was a slight increase in the means of CD4^+^IL-17^+^, CD4^+^IL-22^+^ and CD4^+^IL-17^+^ IL-22^+^ T cells (Supplementary Figure S5).

### 3.7. PrEP and PEP

An analysis of the cohort indicates that individuals with prior exposure to PrEP or PEP exhibited more favorable immunological profiles at the time of PBMC collection compared to those without previous antiretroviral use. Participants with prior PEP use, such as IVA 50 and IVA 70, both classified as Fiebig stage I, demonstrated remarkably low HIV RNA levels (1.84 and 1.59 log copies/mL, respectively) and preserved CD4/CD8 ratios (1.88 and 1.34). Similarly, individuals with previous PrEP exposure, such as IVA 75 and IVA 76, also presented with relatively low viremia (3.39 and 4.00 log). In contrast, participants with no prior ART exposure, particularly those in advanced Fiebig stages (e.g., V), such as IVA 17 and IVA 63, displayed higher viral loads (6.45 and 5.45 log), lower CD4 counts (214 and 403 cells/mm^3^), and severely reduced CD4/CD8 ratios (0.41 and 0.15, respectively). These findings suggest that prior prophylactic antiretroviral exposure may mitigate early immunological damage and contribute to partial control of viral replication during acute HIV infection.

## 4. Discussion

Primary HIV infection represents a critical period and potentially a unique opportunity for ART to induce a profound reduction in the viral reservoir, along with rapid and better immune restoration [37,38]. In this study, we described the virological and immunological parameters of a Brazilian cohort with acute/early HIV infection who started ART during Fiebig stages I–V, focusing on immunopathogenesis and HIV reservoirs in the blood and GALT.

We observed significant immune restoration and viral suppression, with a mean CD4 count of 861 cells/mm^3^, and 92% of participants achieving full viral load suppression at M12 sustained up to M24. In both follow-up visits, nearly all participants reached at least 500 CD4^+^ T cells/mm^3^. Additionally, the use of ART consistently improved the CD4/CD8 ratio, a valuable indicator of overall immune health. These findings are in agreement with previous results showing that very early initiation of ART may lead significant increase in CD4/CD8 ratios [39,40]. Furthermore, a CD4/CD8 ratio ≤1 was associated with a faster time to viral load rebound in a study of early-treated PLWH [41]. Additionally, the D0 CD4/CD8 ratio was the immunological marker which correlated more negatively with viral load levels, and participants with lower Fiebig stages had higher CD4/CD8 ratios. These results are consistent with findings that the initial stage of acute HIV infection, Fiebig I, is associated with more favorable CD4/CD8 ratios compared to the later Fiebig stages II–IV [42]. We observed a strong inverse correlation between HIV-1 DNA and the CD4/CD8 ratio both before ART and after 12 months of treatment, which is in agreement with a study showing the CD4/CD8 ratio as a valuable biomarker for identifying individuals with a smaller HIV reservoir [43].

Measuring HIV-1 DNA levels in PBMCs is a widely used method to assess reservoir size, and it has been associated with time to viral rebound [4,31]. Our findings indicate a notably low frequency of infected cells in individuals with AEHI after 12 and 24 months of ART. This observation may be attributable to the early initiation of ART in these participants, which likely suppressed viral replication to such an extent that the detection of infected cells was beyond the sensitivity of the current assay.

We found a wide variation in total HIV DNA levels among participants, likely due to the immunological and virological variability of this population with different viral load profiles and CD4 count [44,45]. We observed a pronounced decrease in total HIV DNA levels which continued to decline over 24 months after ART initiation. The mean total HIV DNA level in blood cells decreased fourfold at month 12 and sixty-one-fold at month 24 compared to the baseline (D0) visit. These data corroborate the decrease found in a Thai cohort in blood samples suggesting that HIV reservoir is rapidly established in acute HIV infection and dramatically reduced by early ART [46].

Initiating ART at the Fiebig I–III stages has been shown to result in a significant reduction in the frequency of cells with integrated HIV DNA to nearly undetectable levels in all blood, lymph nodes, and colon biopsies by rapidly clearing upon ART initiation [47]. Similarly, we found very low or undetectable levels of HIV DNA in Fiebig I participants. However, we believe this is due to starting ART around the first 72 h after exposure to the virus, which directly influences viral load values and other seroconversion markers and is linked to the Fiebig classification per se. However, what is significant in these data is the fact that undetectable or nearly undetectable HIV in blood and tissues may be associated with prolonged ART-free remission [36,47,48,49].

In addition, we observed concordant results between total HIV DNA levels in PBMC and GALT at D0 among those participants who had both samples available. The concordance between blood and GALT is consistent with other studies [50,51]. In addition, participants with the highest levels of total HIV DNA in GALT exhibited lower CD4^+^ T cell counts. These findings may suggest an association with a compromised immune system, as indicated by the reduced number of CD4^+^ T cells circulating in the blood. This pattern may indicate that the HIV reservoir in GALT is driving a continuous immune activation and depletion of CD4^+^ T cells, reflecting a disrupted balance between viral persistence and immune system dynamics as previously shown [52,53,54,55,56]. Unfortunately, at M12, there were only four tissue samples (75% above the limit of detection) for observing the effects of early ART on immunological parameters associated with the GALT reservoir.

When analyzing the dynamics of immune activation, we observed that early ART use significantly affected the frequency of activated CD8^+^ T cells and slightly impacted activated CD4^+^ T cells in our cohort. This may be related to the important response of CD8^+^ T cells soon after the peak of viremia [57,58]. The activation of CD8^+^ T cells and the expansion of the T cell memory profile were associated with HIV DNA levels, highlighting the importance of viral-host interaction in the size of the viral reservoir [59]. A study with SIV-infected macaques that compared early treatment (4 weeks) with late treatment (24 weeks) found that the early-treated group developed memory CD8^+^ T cells with superior capacity of viral suppression after treatment interruption, suggesting the role of these cells as potential post-treatment control agents [32].

It has been reported that PD-1 expression is elevated during acute HIV infection compared to negative controls [60]. The absence of a negative control group in our study impaired a critical evaluation of the frequency of exhausted cells among the participants. However, we did not observe a reduction in the frequency of exhausted cells after ART. We found that the volunteer with the highest level of exhausted CD8^+^ T cells at D0 also presented higher HIV DNA level after M24, suggesting that early immune dysfunction may result in more persistent viral presence. It is necessary to expand this analysis with more volunteers to confirm this observation. On the other hand, higher HIV DNA levels before the start of ART are associated with greater immune exhaustion at M24, indicating that a higher initial viral reservoir may contribute to greater immune system exhaustion later. Although positive associations between immune exhaustion and HIV reservoir have been previously observed in adults [29,61,62,63] and children [64], we did not find studies describing the bidirectional relationship identified in our data, particularly in acute HIV infection studies.

The functional profile of T cell subsets is profoundly affected by HIV infection, with significant changes in each population. The progressive destruction of CD4^+^ T cells, exhaustion of CD8^+^ T cells, and alterations in regulatory and memory T cells contribute to the complexity of infection control and viral persistence [65,66]. In our cohort, we did not observe any changes in the frequencies of CD4^+^ T cell subsets following treatment. In the CD8^+^ T cell subsets, we observed a different scenario. There was a significant recovery in the frequency of central memory CD8^+^ T cells (TCM) and a decrease in the frequency of effector CD8^+^ T cells (TEF). The reduction in peak viremia alongside the increase in effector CD8^+^ T cell response has already been demonstrated in a population of SIV-infected rhesus macaques [67] and similarly during HIV-1 infection. In the context of acute infection, CD8^+^ T cells emerge in the blood shortly before the peak viral load, expand, and contract as the viral load decreases [44,68]. In chronic infection, high viral load can lead to depletion or impairment of central memory CD8^+^ T cells, compromising the immune system’s ability to respond effectively to HIV and other pathogens [67,69,70]. However, with early ART, viral replication is controlled, reducing the immune system burden, and allowing the maintenance of balance in the number of central memory CD8^+^ T cells [32,57,71,72].

Our analysis also revealed that there were no significant differences in the frequency of pTfh cells (CD4^+^CXCR5^+^) between study visits, prior ART use, or Fiebig stages. This suggests that, in the context of early-treated acute infection, the frequency of pTfh cells may not vary substantially over time or under different treatment conditions. Unfortunately, due to the limited number of cells at D0, we could not evaluate the frequency of Th17 cells at this time point. It has already been demonstrated in humans that Th17 cells are affected at stage III of Fiebig, while they are preserved during stages I/II compared to HIV-negative controls [17]. Considering only the observed means, there is a suggestion of a slight increase in Th17 cell frequencies and the expression of IL-17 and IL-22 between M12 and M24. However, due to data variability, studies with a larger number of samples are needed to confirm or refute these observations and provide a clearer understanding of Th17 cell dynamics during early treatment.

Diagnosing individuals in the very early stages of infection is a significant challenge. In our experience, PrEP and PEP prevention programs have proven to be effective tools for tracking those highly vulnerable and susceptible to HIV. The impact of prophylactic interventions on the HIV reservoir was not the primary focus of this work, largely due to sample size limitations. In our analyses, we were unable to determine any significant influence of antiretroviral use prescribed for PrEP on the parameters evaluated. Nevertheless, given the scarcity of studies investigating seroconversion and acute infection in individuals seeking PrEP, we believe the data presented here may serve as a foundation for future, larger-scale investigations. It is important to note that the very early use of antiretrovirals—whether as PrEP or PEP—directly affects viral replication and immune response, potentially prolonging the stages of seroconversion [73,74]. These factors should be carefully considered when interpreting data in this context. The delay can complicate the diagnosis of HIV infection by extending the typical window period for antibody production. Consequently, more sensitive diagnostic methods, such as HIV viral load, are necessary to confirm HIV status during this time [73,74]. 

The effectiveness of PrEP has been widely confirmed in controlled clinical trials [75,76,77]. However, in everyday practice, PrEP effectiveness has been lower than that observed in clinical trials, primarily due to lower-than-prescribed adherence and frequent discontinuations reported among populations with high vulnerability to HIV [78].

The main highlight of this study is the inclusion of data from a cohort of individuals who were identified and treated early in HIV seroconversion. At the same time, as it is a cohort followed in a routine clinical context, we encountered significant variability in participants’ profiles, including differences in knowledge and access to prevention services, as well as many vulnerable individuals seeking HIV testing for the first time. Several factors interfered with the completion of the study visits, including the COVID-19 pandemic, which led to some participants not undergoing biopsy collection at follow-up visits. This reduced the number of samples available for evaluating reservoirs in GALT at M12 and M24. Additionally, the reduced number of samples at D0 for activation, exhaustion, and subpopulation analyses, along with the lack of samples for Th17 cell analyses and the absence of an HIV-negative control group, limited the ability to thoroughly assess the impact of ART on these markers.

We acknowledge that techniques capable of distinguishing intact from defective proviral DNA offer greater accuracy in reservoir characterization. However, total HIV DNA quantification remains a widely used and accepted method globally. However this limitation must be considered when interpreting our findings and in the formulation of future research. Moreover, proviral DNA was analyzed from bulk PBMCs, without isolating CD4^+^ T cells or monocytes, thereby limiting the ability to determine the specific contribution of each cell population to the HIV reservoir.

## 5. Conclusions

Early initiation of ART during early/acute HIV infection resulted in significant viral suppression and improved immune restoration among study participants. The CD4/CD8 ratio proved to be an important marker for acute viral infection, positively reflecting the effectiveness of the treatment. In general, we observed a positive impact of the early antiretroviral treatment on the levels of viral reservoirs among those previously exposed due to PEP or PrEP prescription. Participants who seroconverted in these scenarios presented with lower levels of HIV DNA. This finding suggests that these prophylactic strategies may play a crucial role not only in preventing HIV acquisition but also in limiting the size of the viral reservoir if infection occurs, if these people are identified early during the period of acute infection or seroconversion. Notably, these are the populations that might benefit the most from strategies aimed at reducing viral reservoirs, such as broadly neutralizing antibodies (bNAbs) and therapeutic vaccines. Presenting a smaller and potentially more manageable viral reservoir may make them ideal candidates for interventions aiming to eliminate the reservoir in further clinical trials.

These findings highlight the importance of initiating ART early and effectively to optimize the immune response and minimize the impact of HIV. Given the evidence of the benefits of ART presented in this work together with the benefits of early diagnosis, strategies to expand PrEP and PEP programs are important for identifying participants in the very early stages of HIV infection. This certainly contributes to more effective control of the HIV pandemic.

## Figures and Tables

**Table 1:** Demographic and clinical characteristics of study participants.

	D0	M12	M24
Age (years), median, [25th–75th]	27 [25–36]		
Gender, *n* (%) Cisgender men Transgender women	23 (95.9%) 1 (4.1%)		
HIV viral load (log_10_ copies/mL; mean [95% CI])	3.9 [3.5–4.3]	<40	<40
CD4 count (cells/mm^3^; mean [95% CI])	646 [540–754]	861 [756–967]	895 [789–1000]
CD4/CD8 (mean [95% CI])	0.75 [0.57–0.94]	1.24 [1.06–1.42]	1.26 [1.08–1.44]
Fiebig Stage (n)			
I	2		
II	1		
III	5		
IV	4		
V	12		

D0 = Day zero or pre-ART visit; M12 = 12 months after treatment; M24 = 24 months after treatment; CI = Confidence interval. I–V: Fiebig stages, see Methods Section (Section 2) for details.

## Data Availability

The data presented in this study is available on request from the corresponding author.

## Supplementary Materials

The following supporting information can be downloaded at: https://www.mdpi.com/article/10.3390/v17101381/s1. Table S1: Baseline clinical and immunological characteristics of participants according to prior use of ART; Figure S1: Gating strategy; Figure S2: Correlations between HIV DNA in PBMC at D0 and HIV DNA in GALT; Figure S3: Correlation matrix of relationships between the parameters analyzed at three visits: before the start of treatment (D0), twelve months after treatment (M12), and twenty-four months after treatment (M24); Figure S4: Longitudinal analysis of the frequency of peripheral follicular helper T cells (pTfh) (T CD4+ CXCR5+); Figure S5: Frequency of Th17 cells (CD4+IL17+) and subsets.
