# Supplementary material for: Acute HIV Infection and ART Response: Insights into T Cell Subsets, Activation, Exhaustion, and Blood and GALT HIV Reservoir
Source: Viruses. 2025 Oct 16;17(10):1381. doi: 10.3390/v17101381 (PMC12568170; doi:10.3390/v17101381)
Supplement: Supplementary file 1 [file viruses-17-01381-s001.zip › viruses-3833992-supplementary.pdf]

viruses

MDPI

Article

Acute HIV Infection and ART Response: Insights into T Cell Subsets, Activation, Exhaustion, and Blood and GALT HIV Reservoir

Soraia Santana de Moura [1], Diogo Gama Caetano [2], Monick Lindenmeyer Guimarães [2], Rayana Katylin Mendes da Silva [2], Natasha Cabral [3], Simone da Costa Cruz Silva [4], Marcelo Ribeiro-Alves [1], Sylvia L. M. Teixeira [2], Ingebourg Georg [5], Desirée Vieira Gomes dos Santos [1], Sandro Nazer [1], Rafael Teixeira Fraga [1], Brenda Hoagland [1], Larissa Villela [1], Beatriz Gilda Jegerhorn Grinsztejn [1], Valdiléa Gonçalves Veloso [1], Fernanda Heloise Côrtes [2,*,†] and Sandra W. Cardoso [1,*,†]

1 Laboratório de Pesquisa Clínica em IST e AIDS, Instituto Nacional de Infectologia Evandro Chagas, Fundação Oswaldo Cruz, Rio de Janeiro 21040-900, Brazil; soraia.moura@ini.fiocruz.br (S.S.d.M.); marcelo.ribeiro@ini.fiocruz.br (M.R.-A.); desiree.santos@ini.fiocruz.br (D.V.G.d.S.); sandro.nazer@ini.fiocruz.br (S.N.); brenda.hoagland@ini.fiocruz.br (B.H.); laramvillela@gmail.com (L.V.); gbeatriz@ini.fiocruz.br (B.G.J.G.); valdilea.veloso@ini.fiocruz.br (V.G.V.)

2 Laboratório de Aids e Imunologia Molecular, Instituto Oswaldo Cruz, Fundação Oswaldo Cruz, Rio de Janeiro 21040-900, Brazil; diogocaetano@aluno.fiocruz.br (D.G.C.); monicklg@ioc.fiocruz.br (M.L.G.); sylvia@ioc.fiocruz.br (S.L.M.T.)

3 Laboratório de Hanseníase, Instituto Oswaldo Cruz, Fundação Oswaldo Cruz, Rio de Janeiro 21040-900, Brazil; natasha.cabral@ioc.fiocruz.br

4 Plataforma de Laboratório Multiusuário, Instituto Nacional de Infectologia Evandro Chagas, Fundação Oswaldo Cruz, Rio de Janeiro 21040-900, Brazil; simone.silva@ini.fiocruz.br

5 Laboratório de Imunologia, Instituto Nacional de Infectologia Evandro Chagas, Fundação Oswaldo Cruz, Rio de Janeiro 21040-900, Brazil; ingebourg.georg@ini.fiocruz.br

* Correspondence: fernanda.cortes@fiocruz.br (F.H.C.); sandra.wagner@ini.fiocruz.br (S.W.C.)

† These authors contributed equally to this work.

 https://doi.org/10.3390/v17101381

Supplementary Table S1: Baseline clinical and immunological characteristics of participants according to prior use of ART

ID	Previous use of ART	Prior use of ART before PBMC collection (D0)	FIEBIG Stage	HIV RNA at D0 (log/copies/mL)	CD4 at D0 (cells/mm³)	CD4/CD8 at D0
IVA 17	none	0	V	6.45	214	0.41
IVA 23	none	0	V	5.15	715	0.44
IVA 30	none	1	V	4.61	348	0.91
IVA 34	none	0	V	6.58	675	0.33
IVA 35	none	0	V	5.05	896	0.59
IVA 36	none	0	IV	6.13	854	0.57
IVA 37	none	0	IV	6.30	1067	0.22
IVA 38	none	0	III	5.65	485	0.95
IVA 41	none	0	IV	4.81	593	0.41
IVA 54	none	0	V	4.72	NA	NA
IVA 56	none	0	IV	3.01	1002	0.72
IVA 57	none	0	V	7.36	535	0.21
IVA 61	none	0	V	4.51	584	0.5
IVA 63	none	0	V	5.45	403	0.15
IVA 79	none	0	V	2.30	482	0.66
IVA 82	none	0	V	7.00	221	0.28
IVA 87	none	0	III	7.00	498	0.76
IVA 84	none	0	III	5.19	608	1.16
IVA 75	PrEP	0	V	3.39	539	0.76
IVA 76	PrEP	0	III	4.00	724	1.77
IVA 50	PEP	8	I	1.84	1092	1.88
IVA 62	PEP	4	III	5.80	933	1.65
IVA 32	PEP	4	II	4.58	574	0.93
IVA 70	PEP	12	I	1.59	508	1.34

ID – Participant Identification Number; ART – Antiretroviral Therapy; PBMC – Peripheral Blood Mononuclear Cells; D0 – Day of PBMC Collection (Baseline); PrEP – Pre-Exposure Prophylaxis; PEP – Post-Exposure Prophylaxis; NA – Not Available.

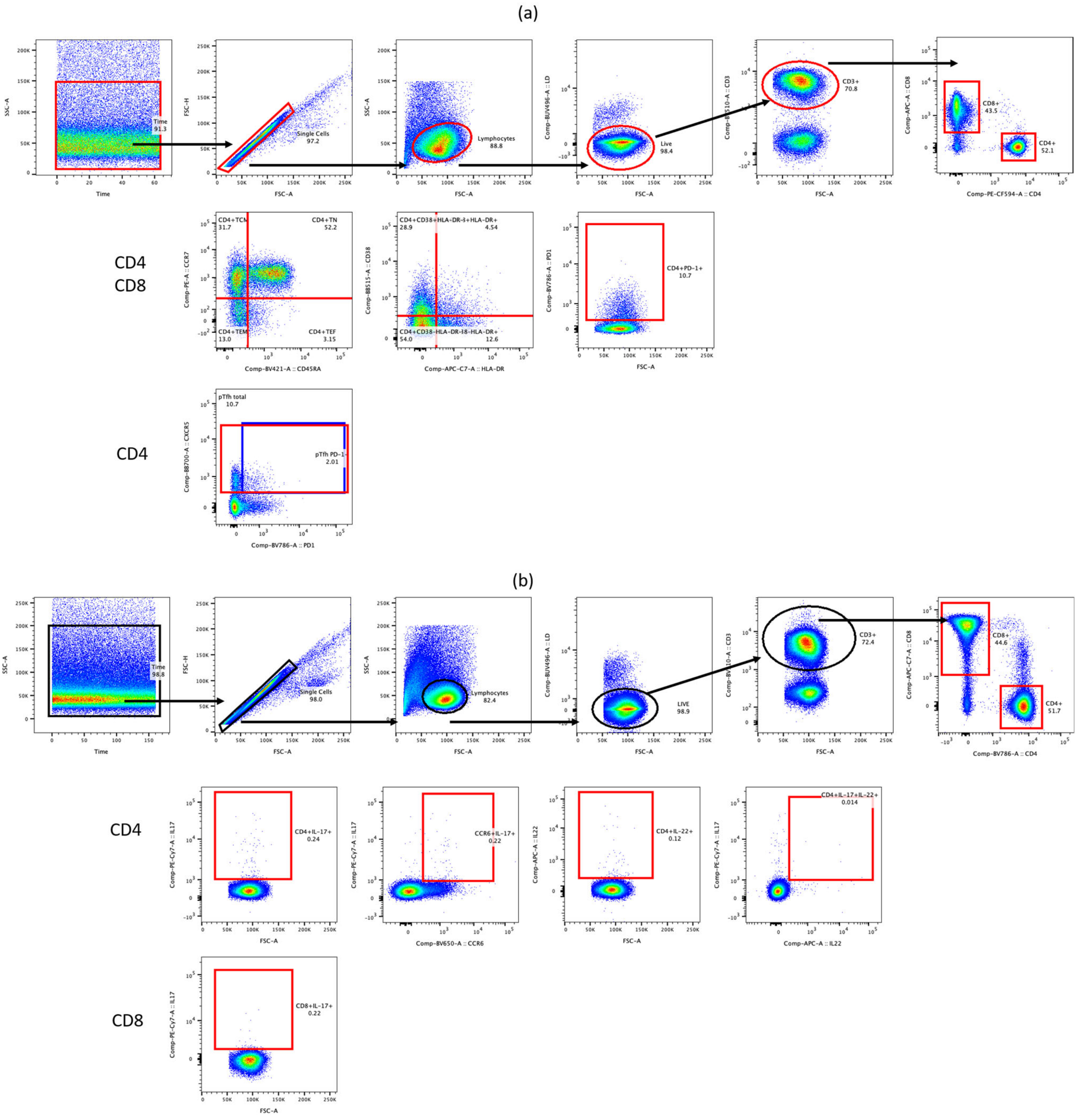

Supplementary Figure S1: Gating strategy: (a) Gating strategy for memory T cell subsets, activated and exhausted T cells, and pTfh cells. Cells were sequentially gated as follows: time > single cells > lymphocytes > live cells > CD3⁺ T cells > CD4⁺ and CD8⁺ T cells. Within the CD4⁺ and CD8⁺ T cell populations, CCR7 vs CD45RA was used to identify naïve, central memory, effector memory, and effector subsets; CD38 and HLA-DR were used to identify activated cells (CD38 vs HLA-DR); and PD-1 vs FSC-A was used to identify exhausted cells. pTfh cells were gated within the CD4⁺ T cells using a CXCR5 vs PD-1 dot plot, where all CXCR5⁺ cells were defined as pTfh cells. (b) Gating strategy for Th17 and Tc17 cells. Cells were sequentially gated as follows: time > single cells > lymphocytes > live cells > CD3⁺ T cells > CD4⁺ and CD8⁺ T cells. Within the CD4⁺ T cells, Th17-related subsets were gated as follows: IL-17⁺ (IL-17 vs FSC-A), IL-22⁺ (IL-22 vs FSC-A), CCR6⁺IL-17⁺ (CCR6 vs IL-17), and IL-17⁺IL-22⁺ (IL-17 vs IL-22). Tc17 cells were gated within the CD8⁺ T cells based on IL-17 expression (IL-17 vs FSC-A).

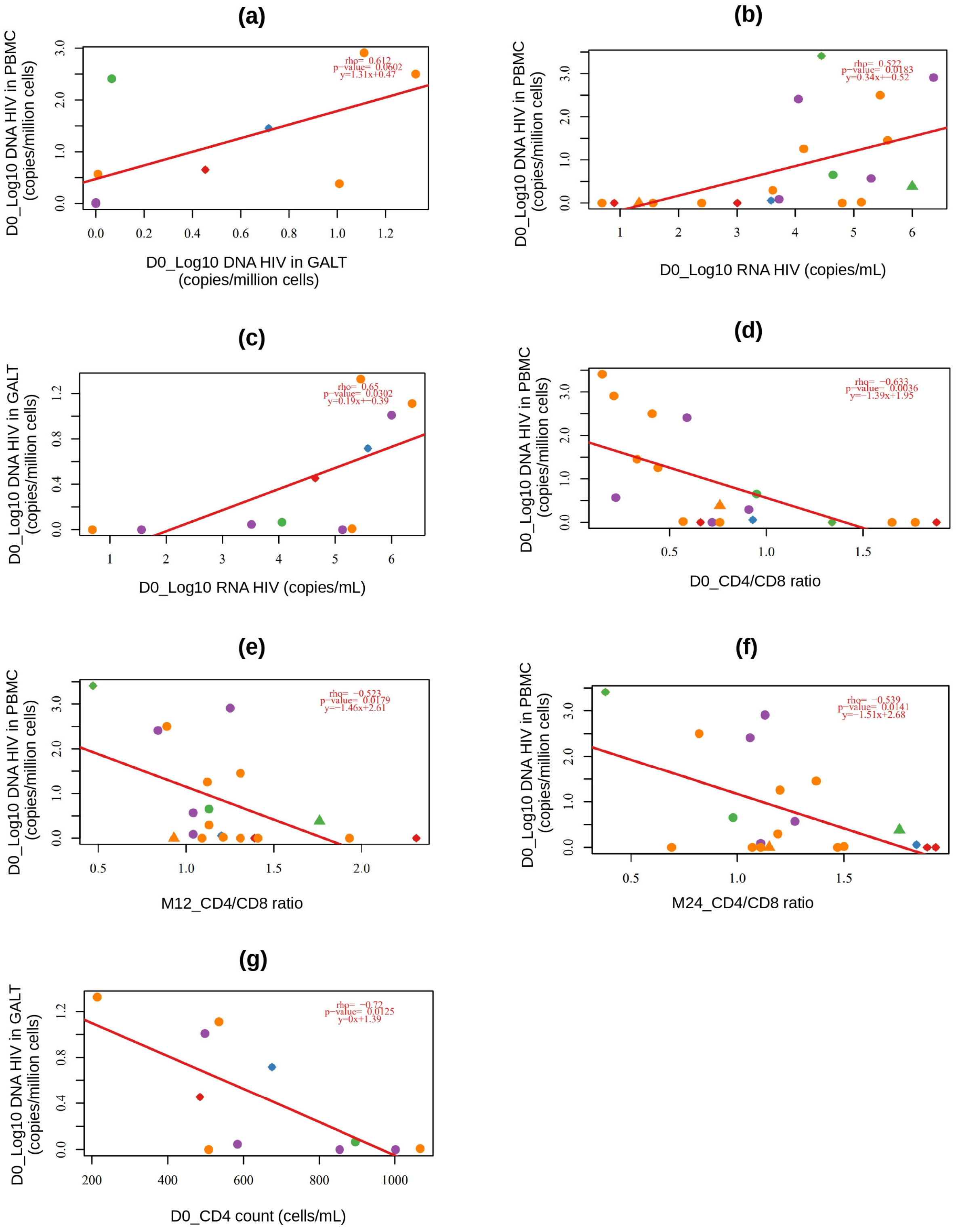

Supplementary Figure S2: Correlations between HIV DNA in PBMC at D0 and HIV DNA in GALT (a), plasma HIV viral load (b), CD4/CD8 ratio at D0 (d), CD4/CD8 ratio at M12 (e), and CD4/CD8 ratio at M24 (f). Correlations between HIV DNA in GALT at D0 and plasma HIV viral load (c) and CD4 T cell count (g). Correlations were analyzed using Pearson's correlation test.

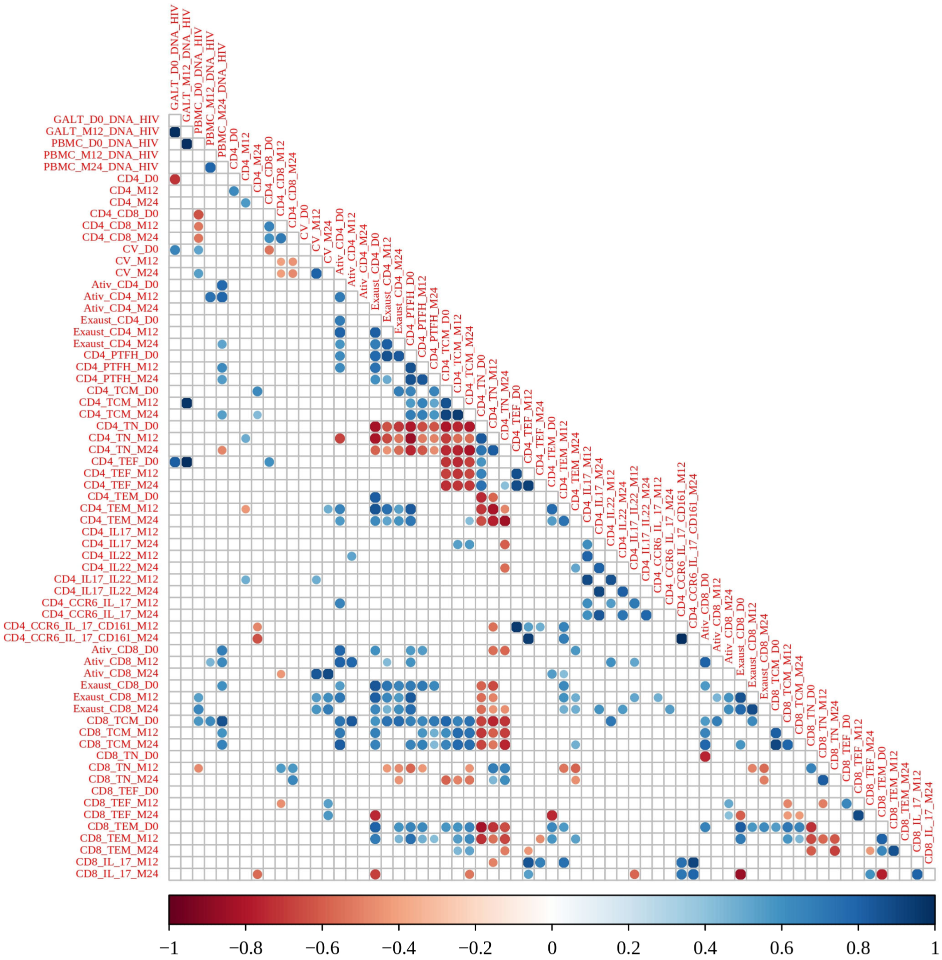

Supplementary Figure S3: Correlation matrix of relationships between the parameters analyzed at three visits: before the start of treatment (D0), twelve months after treatment (M12), and twenty-four months after treatment (M24). Correlations were then calculated using Pearson's correlation test. The circle size and color intensity represent the strength of the correlations as indicated in the bottom color bar. Only significant p-values ($p < 0.05$) are represented in the figure.

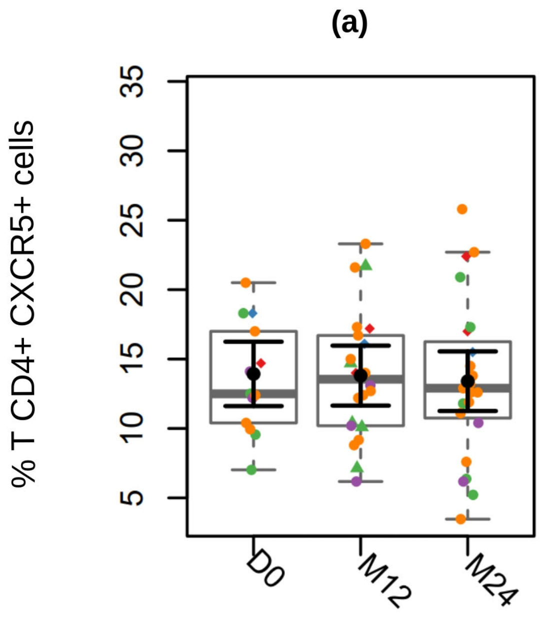

Supplementary Figure S4: (a) Longitudinal analysis of the frequency of peripheral follicular helper T cells (pTfh) (T CD4+ CXCR5+). Fiebig stages are identified by colors as follows: red (I), blue (II), green (III), purple (IV), and orange (V). Participants who began ART prescribed as post-exposure prophylaxis (PEP) before the diagnosis are represented by the diamond symbol, while those identified while seeking pre-exposure prophylaxis (PrEP) at the time of diagnosis are represented by triangle symbol. For black error bars, central dots represent mean marginal estimates and parallel lines indicate the upper and lower 95 % confidence interval limits. Grey boxplots represent the boxplot of the sampled distribution. P-values were generated from mixed linear regression analysis and correlations were analyzed using Pearson's correlation test.

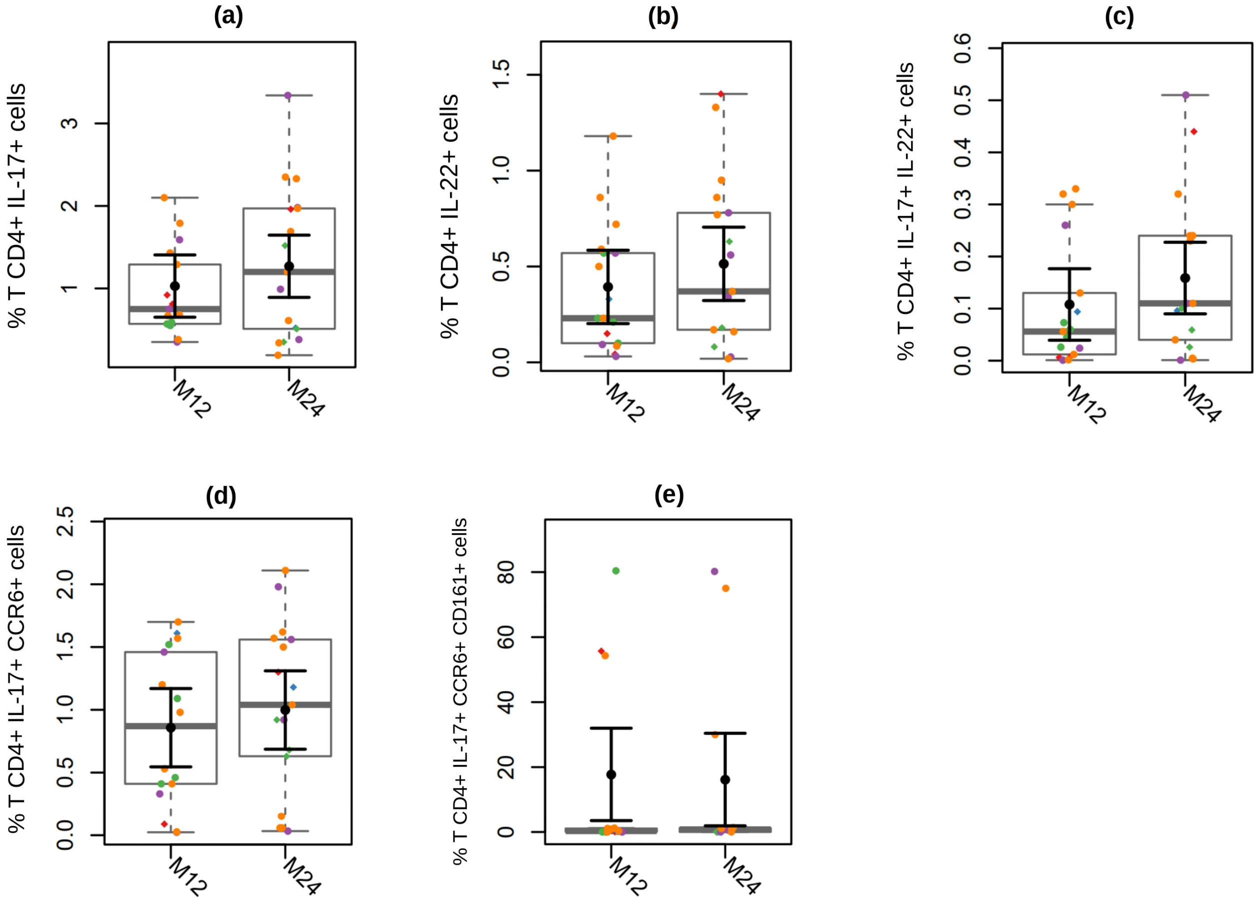

Supplementary Figure S5: Frequency of Th17 cells (CD4+IL17+) and subsets. Longitudinal analysis of the frequency of expression of Th17 cells by the markers (a) IL-17+, (b) IL-22+, (c) both IL-17+ and IL-22+, (d) IL-17+ CCR6+, and (e) IL-17+ CCR6+ CD161+. Fiebig stages are identified by colors as red (I), blue (II), green (III), purple (IV), and orange (V). Participants who began ART prescribed as post-exposure prophylaxis (PEP) before the diagnosis are represented by the diamond symbol, while those identified while seeking pre-exposure prophylaxis (PrEP) at the time of diagnosis are represented by triangle symbols. For black error bars, central dots represent mean marginal estimates and parallel lines indicate the upper and lower 95 % confidence interval limits. Grey boxplots represent the boxplot of the sampled distribution. p-values derived from mixed linear regression analysis.
